# Durable antibody and effector memory T cell responses in breastmilk from women with SARS-CoV-2

**DOI:** 10.3389/fimmu.2022.985226

**Published:** 2022-09-12

**Authors:** Vignesh Narayanaswamy, Brian T. Pentecost, Janice C. Telfer, Amy S. Burnside, Sallie S. Schneider, Dominique Alfandari, Ryan L. Baker, Aman Saiju, Sam Nodiff, Kathleen F. Arcaro

**Affiliations:** ^1^ Department of Veterinary and Animal Sciences, University of Massachusetts, Amherst, MA, United States; ^2^ Pioneer Valley Life Sciences Institute, Baystate Medical Center, Springfield, MA, United States

**Keywords:** breast milk, COVID-19, SARS-CoV-2, flow cytometry, memory T cells, mucosal immunity, breast permeability

## Abstract

**Background:**

Given that only 25% of pregnant women elect to receive a COVID-19 vaccine, maternal SARS-CoV-2 infection remains an important route of conferring protective passive immunity to breastfed infants of mothers who are not vaccinated.

**Methods:**

We enrolled 30 lactating participants between December 2020 and March 2021 who had a positive PCR-test and their first COVID-19 symptoms within the previous 21 days. Participants were asked to provide serial bilateral milk samples at 12 timepoints (~ every 3 days) over a period of 35 days. A second set of samples was collected at least four months after the beginning of the first set. Participants also were asked to provide their dried blood spots and infant stool samples. All samples were tested for receptor-binding domain (RBD)-specific immunoglobulin (Ig)A, IgG, and IgM. Milk samples were assessed for neutralizing ability against the spike protein and four SARS-CoV-2 variants: D614G, Alpha (B.1.1.7), Beta (B.1.351), and Gamma (P.1). Permeability of the breast epithelium was assessed by measuring the sodium to potassium ions (Na:K) in milk. Using flow cytometry, memory CD4 and CD8 T cells (CD45RO^+^ and CCR7^+/-^) and mucosal-homing CD4 and CD8 T cells (CD103^+^) were determined in cells from milk expressed at 35 days and at least 4 months after their first milk donation.

**Results:**

Milk antibodies from SARS-CoV-2 positive participants neutralized the spike complex. Milk from 73, 90, and 53% of participants had binding reactivities to RBD-specific IgA, IgG, and IgM, respectively. In contrast to blood spots, which showed increased levels of IgG, but not IgA or IgM, the COVID-19 response in milk was associated with a robust IgA response. Twenty-seven percent of participants had increased breast-epithelium permeability, as indicated by Na:K ≥ 0.6. The percentage of CD45RO^+^CCR7^-^ effector-memory T cells in the day ≥120 milk samples was significantly higher than day 35 samples (*P*< 0.05).

**Conclusions:**

Antibodies in milk from participants with recent SARS-CoV-2 infection and those who recovered can neutralize the spike complex. For the first time we show that breastmilk T cells are enriched for mucosal memory T cells, further emphasizing the passive protection against SARS-CoV-2 conferred to infants *via* breastmilk.

## Introduction

The American College of Obstetricians and Gynecologists, and the Academy of Breastfeeding Medicine recommend that breastfeeding women receive any WHO-approved COVID-19 vaccine (1, 2), however, only 25% of pregnant women elect to receive the vaccine (3). Therefore, as we and others have shown, maternal SARS-CoV-2 infection remains an important route of conferring protective passive immunity to breastfed infants of mothers who are not vaccinated (4–10). We previously reported on 30 bilateral colostrum samples obtained in 2020 from 15 unvaccinated women who either had active infections or had recently recovered from COVID-19 (9). In that study, colostrum from 73% of the women exhibited binding reactivities to the receptor binding domain (RBD) of SARS-CoV-2. We also found that colostrum from symptomatic COVID-19 women had significantly elevated levels of IFN-γ, IL-4, IL-6, and IL-12. We also examined the time course of the immune response in milk from women who received an mRNA vaccine (11). While the immune response in milk of women with COVID-19 was both IgA and IgG, the immune response in milk of women who received a vaccine was primarily IgG. Interestingly both COVID-19 and vaccination induced significant increases in cytokine levels in milk. Assessing levels of cytokines in milk is important as these proteins can affect the development of the infant gut and immune system (12, 13).

In our previous studies, we did not examine the immune cells in milk. It is known that the phenotypic T cell response to infection can differ significantly between milk and peripheral blood (14, 15). Studies using animal models suggest that breastmilk T cells can survive in the infant gastrointestinal tract and traffic to the liver, mesenteric lymph nodes, and the spleen (16–18). These data highlight the potential for milk-derived T cells to confer passive protection to infants. However, the phenotypic immune response to COVID-19 infection in breastmilk has not been described.

To the best of our knowledge, we are the first to assess the phenotypic immune response to COVID-19 infection in breastmilk. We used flow cytometry of cryopreserved cells from milk provided around 35 days and four months after a positive COVID-19 test to determine the levels of memory markers using CD45RO and CCR7 staining, and mucosal-homing markers using CD103. We also assessed the levels of anti-RBD immunoglobin (Ig)A, IgG and IgM in serial milk samples provided over 34 days and in an additional milk sample expressed more than four months after collection of the initial frozen milk samples. Finally, we examined milk for changes in levels of ten cytokines and breast permeability, and infant stool for levels of anti-RBD IgA, IgG and IgM.

## Methods

### Recruitment of participants

Recruitment information was posted on the breastmilkresearch.org website. Individuals from across the continental U.S. could enroll if they were lactating and had their first COVID-19 symptoms within the previous 21 days, with a positive PCR test. Although exclusive breastfeeding is recommended for the entire window covered by the serial milk-collection-period of our study, exclusive breastfeeding was not a requirement for participation. A total of 30 lactating women were enrolled in the study and provided consent under UMass Amherst IRB-approved protocol 2075. After enrollment, women were asked to complete a demographic and general health questionnaire for themselves and their infant, and the date of their COVID-19 positive test. All information was logged *via* Research Electronic Data Capture (REDCap). Note: two enrolled women delayed collecting their first milk sample; the time between their COVID-19 positive test and first milk collection was greater than 21 days (P053, first sample collected 28 days after positive COVID-19 test, and P068, first sample collected 33 days after positive COVID-19 test; **Table S1**).

### Sample collection

**Figure 1:**
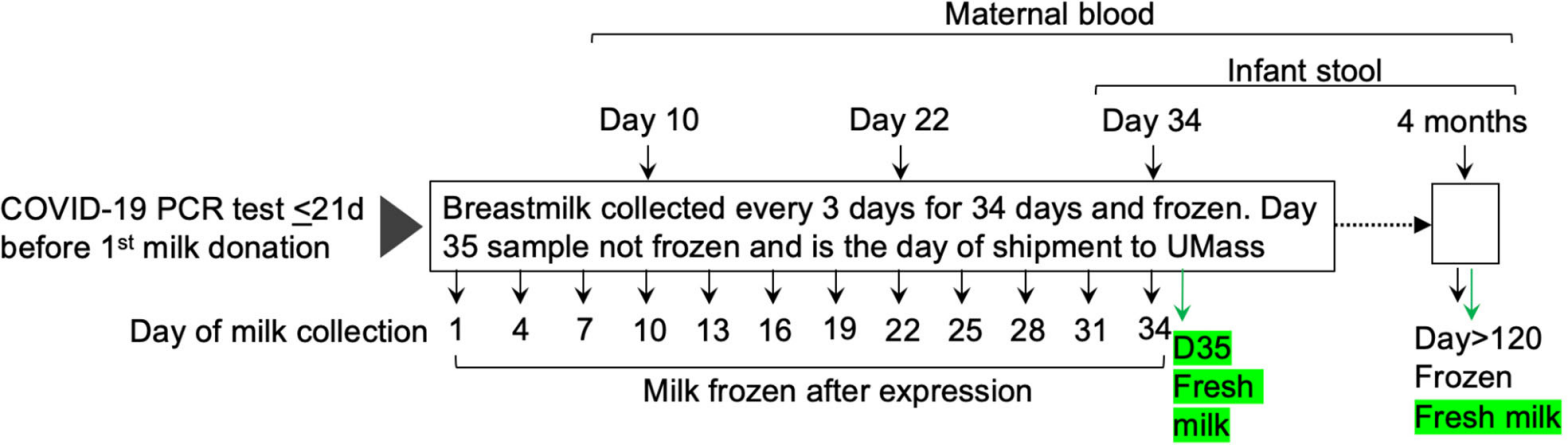
Overview of collection of milk, dried blood spot cards, and infant stool samples. Thirty lactating participants who had a positive PCR-test and their first COVID-19 symptoms within the previous 21 days were enrolled between December 2020 and March 2021. Participants were asked to provide serial bilateral milk samples at 12 timepoints (~ every 3 days) over 35 days. A second set of samples was collected at least four months after the first sample from the first set. Participants also were asked to provide their blood spotted onto cards and samples of their infants’ stool at the indicated timepoints. Fresh, never previously frozen, milk samples (indicated in green) were obtained at the two indicated timepoints.

Consented participants were sent kits with instructions for sample collection, storage, and return shipping for two distinct sets of samples (**Figure 1**). For the first set of samples, participants were asked to provide serial milk samples from each breast (bilateral breastmilk samples) at 12 timepoints (~ every 3 days) over 34 days. At each timepoint, participants completed a questionnaire, *via* REDCap, about the milk collection and SARS-CoV-2-related symptoms for themselves and their infant. Participants were instructed to freeze their milk at each timepoint until all samples were collected and were ready to be shipped to UMass Amherst. Participants also were asked to provide a sample of their blood collected at three time points (10, 22, and 34 days after their first milk sample), and an infant stool sample (collected at day 34) using the provided kits as previously described (11). Briefly, bloodspots were collected on cards (Whatman FTA card, #WHAWB120205) and left to dry at room temperature (RT) (dried blood spots; DBS), and infant stool samples were collected in stool-collection tubes (Fisher Sci., Cat. No. NC0705093) containing 8 mL of 95% ethanol, and on Fecal Occult Blood Test cards (not used in the present study). On the day of the first shipment, bilateral milk samples were collected and not frozen (these milk samples were used for flow cytometry analysis). Frozen and fresh milk, maternal blood spots, and infant stool samples were shipped with ice packs to UMass Amherst *via* overnight courier.

The second shipment occurred at least four months after the mothers provided their initial frozen milk samples and included bilateral frozen and fresh milk, maternal blood spots, and infant stool. Collection and shipment methods were the same as those used for the shipment on day 35.

### Processing breastmilk, DBS, and infant stool samples for immunoassays

All samples were processed as described previously (11). Briefly, equal volumes of bilateral milk samples were mixed to generate a combined sample. Five hundred microliters of combined milk were centrifuged at 820xg for eight minutes at RT. The whey fraction was carefully transferred to a 48-well plate, and samples from the plate were used for the detection of SARS-CoV-2 RBD-specific immunoglobulins and cytokines, and in the neutralization assay.

Discs (6 mm diameter) were prepared from DBS cards and transferred to 24-well plates. Five hundred microliters of TBST (Tris-buffered saline with 0.05% Tween 20) were added to the DBS discs, and the plate was incubated with gentle shaking overnight at 4°C. Blood spot eluates were used for the detection of SARS-CoV-2 RBD-specific immunoglobulins.

Infant stool samples received in stool-collection tubes containing 95% ethanol were vortexed for 20 minutes until a homogenous suspension was achieved. Aliquots of the stool samples were prepared and stored at -20°C. A single aliquot was retrieved at the time of analysis and centrifuged at 4,000xg for 20 minutes at 4°C. After centrifugation, the ethanol supernatant was aspirated, leaving behind the stool pellet, to which 1 mL of TBST was added. The tube was vortexed for five minutes, centrifuged at 4,000xg for 20 minutes at 4°C, and the TBST supernatant was used for the detection of total and SARS-CoV-2 RBD-specific immunoglobulins in infant stool.

### Enzyme-linked immunosorbent assay (ELISA) for detection of total and SARS-CoV-2-specific immunoglobulins

SARS-CoV-2 RBD-specific and total immunoglobulins were measured as previously described using enzyme-linked immunosorbent assays developed and validated at UMass Amherst (9, 11). The RBD on the virus spike protein is involved in docking of virus to cells, elicits a clear immune response, and is the assayed target in most commercial screening tests.

### Antibody neutralization assay

The neutralization assay was performed using a V-PLEX SARS-CoV-2 Panel 6 multiplex assay by Mesoscale Discovery (K15436U). The assay quantitatively measures antibodies in the sample that can inhibit the interaction of spike and its variants with ACE2. Each plate included 8 standards. We are reporting results for wildtype spike protein (referred to as spike) and four spike variants: D614G, B.1.1.7 (Alpha), B.1.351 (Beta), and P.1 (Gamma). All samples and standards were run in technical duplicates. We compared neutralizing ability in milk provided at three timepoints: the first milk samples expressed after enrollment (n = 30, Day 1), and the last of the serial milk samples expressed after enrollment (n = 30, Day 34), and samples collected at the four-month follow-up (n = 15, Day≥120).

### Cytokine assay

Cytokines were measured in a multiplex assay (Mesoscale Discovery) according to the manufacturer’s instructions using 10-plex human V-PLEX Proinflammatory Panel 1 plates (K15049D), as previously described (11). Each 96-well includes assays for 10 cytokines: interleukin (IL)-2, IL-4, IL-6, IL-8, IL-10, IL-12p70, IL-13, IL-1β, interferon (IFN)-γ, and tumor necrosis factor (TNF)-α. Each plate included eight standards and 40 samples in technical duplicates. Cytokines were measured in aliquots of the same samples that were used in the neutralization assay.

### Flow cytometry

Cells from milk expressed at day 35 and at least four months after the first milk donation (Day≥120) and shipped to the lab fresh (never frozen) were cryopreserved for flow cytometry upon receipt at the lab. The milk was diluted with equal volume of 1X phosphate buffered saline (PBS) and centrifuged at 820xg for eight minutes at RT. The supernatant was discarded, and the cell pellet was washed with 1X PBS. The cell suspension was centrifuged at 420xg for eight minutes. The cell pellet was resuspended in 1 mL of freezing media (45% fetal calf serum, 45% Dulbecco’s modified essential medium, and 10% dimethyl sulfoxide) and cryopreserved for flow cytometry.

For flow cytometry, cryopreserved milk cells were thawed and washed with FACS buffer (1X PBS + 2% bovine serum albumin). Cells were incubated for ten minutes at room temperature in 5 μL of 0.5 mg/mL Fc-block (BD Pharmingen) and subsequently stained with 100 μL of master mix composed of the optimal dilutions of fluorophore-conjugated antibodies (BioLegend) (**Table S2**). Cells were stained for 20 minutes at 4°C, protected from light. Cells were then washed with 1 mL of FACS buffer, and the cell pellet was resuspended in 100 μL of FACS buffer for flow cytometry analysis. Cells were run on BD LSR Fortessa and the following cell populations were assessed: CD3 (PE/Cy7), CD4 (FITC), CD8 (PerCP/Cy5.5), CD103 (BV421), CD45RO (PE), CCR7 (APC). Single stained compensation beads (Cat. No. B22804, Beckman Coulter) served as compensation controls, and fluorescence-minus one (FMO) stained cells were used to set fluorescence gates. CD103^+^ cells on both CD4 and CD8 populations were defined as mucosal-homing T cells. CD45RO^+^/CCR7^+^ were classified as central memory T cells (T_CM_) and CD45RO^+^/CCR7^-^ were effector memory T cells (T_EM_). The gating strategy is shown in **Figure S1**. The data were analyzed in FlowJo version 10.8.

### Measurement of sodium to potassium ions (Na:K)

Sodium and potassium levels were measured using ion-selective electrode probes (Medica EasyLyte Na/K Analyzer). Briefly, 1 mL aliquots of whole milk were thawed, centrifuged at 320xg for three minutes at RT and the sodium and potassium concentrations determined in the clarified whey fraction. The Na:K ratios are used as an indicator of increased breast epithelium permeability due to leaky tight junctions (19).

### Statistical analysis

Participant characteristics with continuous outcome measures are reported as mean and range, and characteristics with categorical outcomes are reported as percentages. The thresholds for positivity for RBD-specific antibodies were set at optical density values three times above the SD of the optical density values obtained with only the secondary antibody (background) (11), and samples with ODs ≥ 0.5 were considered high. For the neutralization assay, the concentration of antibodies that inhibited the binding of ACE2 to the spike or its variants (percent inhibition) was computed using the equation provided by the manufacturer $(1-\frac{Average\;Sample\;ECL\;signal}{Average\;ECL\;signal\;of\;the\;blank})\;\times \;100.$ Independent t-tests were used to analyze differences in RBD antibodies, percent inhibition of neutralizing antibodies, mucosal-homing, and effector memory markers between control and COVID-19 groups and as a function of time. Pearson R was used to determine the correlation between RBD-antibodies and neutralizing ability. *P*< 0.05 was considered statistically significant. Statistical analyses were performed using GraphPad Prism 9.

## Results

### Participant demographics

Demographic characteristics and COVID-19 related information are provided in **Table 1**. The study population consisted of women who self-identified as White (*n* = 27), Latina (*n* = 1), or Asian (*n* = 2) and their breastfeeding infants. At enrollment, women were between 25 and 40 years of age (mean = 32 years) and their infants were between 9 and 150 days old (mean = 87 days). Milk samples were collected at the timepoints indicated in **Figure 1** by 30 lactating women beginning 1 to 33 days after a positive PCR test for SARS-CoV-2. Twenty-eight women provided bilateral samples, one woman provided milk samples combined from the left and right breasts, and another woman provided milk from either or both breasts. Only 15 women (50%) provided milk at the four-month timepoint.

**Table 1:** Participant demographics and COVID-19 related information (*n* = 30).

Characteristics	
	**Mean (Range)**
Mother’s Age (year)	32 (25-40)
Baby’s Age (Days)	87 (9-150)
BMI (kg/m^2^)	28 (20-40)
	***n* (%)**
**Race**	
White	27 (90)
Latino	1 (3)
Asian	2 (7)
**Number of women who provided milk**	
Serial frozen samples over 34 days	30 (100)
Day 35 fresh sample	18 (60)
Day≥120 frozen sample	15 (50)
Day≥120 fresh sample	18 (60)
**Number of women who provided DBS cards**	24 (80)
Sample 1^a^	24 (80)
Sample 2^b^	21 (70)
Sample 3^c^	18 (60)
Sample 4^d^	11 (37)
**Infant stool samples provided**	26 (87)
Sample 1^e^	25 (83)
Sample 2^f^	11 (37)
**Maternal COVID-19 related symptoms**	
Fever symptoms	2 (7)
Tiredness	14 (47)
Aches	4 (13)
Runny nose	8 (27)
Sore throat	3 (10)
Headache	3 (10)
Cough	7 (23)
Shortness of breath or difficulty breathing	3 (10)
Loss of sense of smell	11 (37)
Loss of sense of taste	16 (53)
No symptoms	5 (17)
**Infant symptoms**	
Fever symptoms	1 (3)
Runny nose	4 (13)
Cough	3 (10)
Vomiting	1 (3)
No symptoms	22 (73)

Blood spots obtained 10^a^, 22^b^, 34^c^, and ≥120^d^ days after participants provided their initial frozen bilateral milk. Infant stool obtained 34^e^ and ≥120^f^ days after participants provided their initial frozen milk samples.

Maternal and infant symptoms related to COVID-19 are summarized in **Table 1**. The symptoms reported in **Table 1** were recorded at the time women collected their first milk sample, which ranged from 3 to 33 days (mean = 14 days) after a positive COVID-19 test, and at each subsequent milk collection. Overall, 83% of the women reported experiencing one or more COVID-19 related symptoms; the most common being loss of sense of taste. When infant symptoms did occur, they included fevers (3%), runny nose (13%), cough (10%) and/or vomiting (3%). However, most women (73%) reported that their infants had no symptoms.

### The levels of functional SARS-CoV-2-specific antibodies detected in breastmilk vary among women

**Figure 2:**
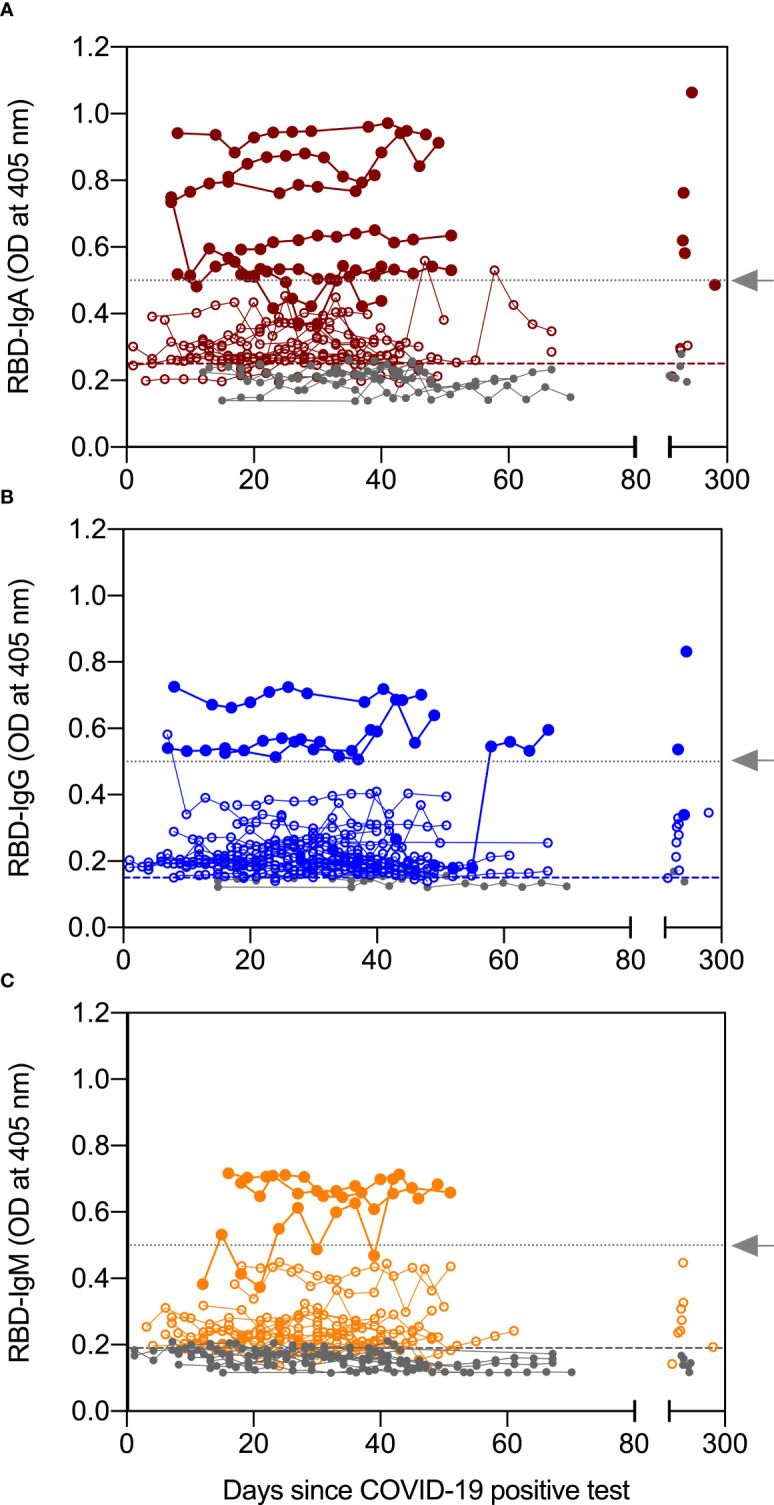
Antibody levels in serial milk samples after SARS-CoV-2 infection. Serial milk samples were obtained across 34 days after a woman enrolled in the study, which was within 21 days of her symptoms and positive COVID-19 PCR test (except for 2 women, see text for details). A final sample was obtained ≥120 days after the initial milk sample. The X axis shows the sample collection for all 30 women relative to their positive COVID-19 PCR test; day 0 indicates day of positive COVID-19 test. Whey fractions were assessed with ELISA for receptor binding domain (RBD)-specific immunoglobulin (Ig)A **(A)**, IgG **(B)**, and IgM **(C)**. Horizontal dashed lines indicate the positive cut off values and horizontal dotted lines (grey arrow) indicate an OD of 0.5. Levels greater than 0.5_OD_ are grouped into high responders.

In our previous studies, we showed, using archived pre-pandemic milk samples from 20 women, that median levels of RBD-reactive antibodies were below positive cut-off limits and had minimal cross-reactivity (data not shown) (11, 20). In the present study, we measured anti-RBD IgA, IgG, and IgM in serial milk samples provided by 30 women who had a positive test by PCR for SARS-CoV-2 (**Table S1**). Serial milk samples from 22 of 30 women (73%) were positive for anti-RBD IgA. At D≥120, milk from nine of the 15 (60%) women who provided samples were positive for anti-RBD IgA, a decrease of 13%. Four of the IgA positive, D≥120 samples belonged to the high group defined as ODs ≥ 0.5 (**Figure 2A**). **Figure 2B** shows that 27 of 30 (90%) women had anti-RBD IgG above the positive cut-off value, with levels remaining positive in milk obtained D≥120 from all but one woman. Fifty-three percent of women tested positive for anti-RBD IgM following their positive COVID-19 test and 46% of women who provided milk during the four-month follow-up had positive RBD-IgM levels (**Figure 2C**). Although more women had IgG responses in their milk compared to IgA (90% vs 73%), the number of women having a high response, as defined by OD ≥ 0.5, was greatest for IgA (filled circles in **Figure 2**).

In contrast to milk, serum humoral response to SARS-CoV-2 infection was primarily IgG. Twenty-four women provided DBS samples collected around ten days after their COVID-19 positive test (**Table 1**). The blood of all women contained RBD-specific IgG with levels remaining positive across all timepoints of collection, while median levels of RBD-specific IgA and IgM in the DBS were below the assays’ positive cut-off for most samples (**Figure S2**).

**Figure 3:**
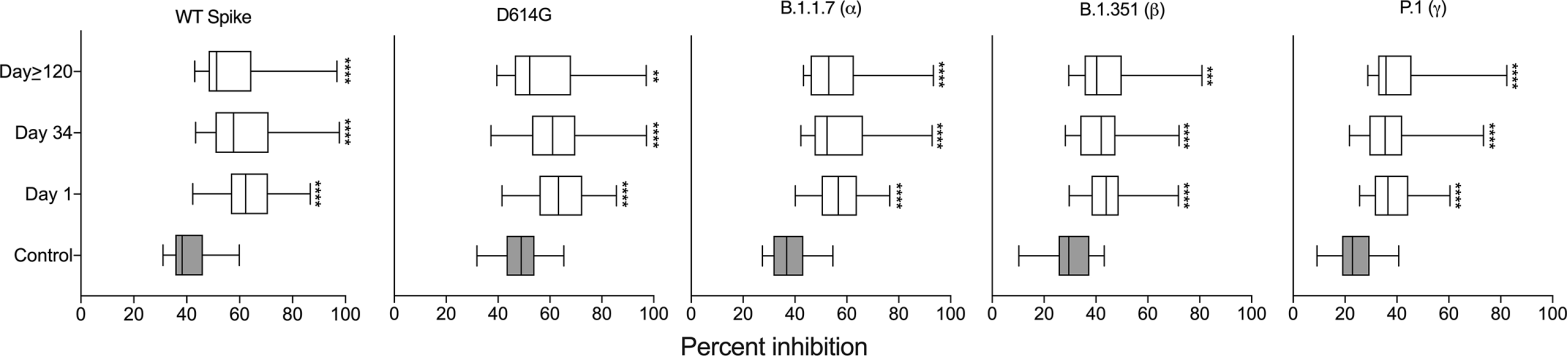
Milk samples from SARS-CoV-2-infected women have neutralizing capacity against the viral spike complex. Comparison of percent inhibition of spike and its variants between control and milk provided at the three indicated timepoints; independent *t*-tests ****P < 0.0001. The control data are from our previous publication (11) in which women who had not had COVID-19 provided milk prior to vaccination. **P<0.01; ***P<0.001.

We next assessed the neutralizing ability of SARS-CoV-2-elicited antibodies in milk. We chose three time points for our analysis: the first milk sample provided = Day 1 (collected 3-33 days after a positive PCR test), the last milk sample in the first set termed Day 34 (collected 36-68 days after the Day 1 sample collection), and the milk sample provided at least four months after the Day 1 sample = Day≥120 (collected 123-253 days after the Day 1 sample collection). Antibody neutralization is reported as percent inhibition of binding by purified ACE2 to immobilized targets, either spike or one of the four variants (D614G, alpha, beta, and gamma). As a control we included the median percent inhibition of milk provided by women without COVID-19 from an earlier study (11). These samples allowed us to establish baseline percent inhibition for milk. Compared to controls, milk from women with COVID-19 significantly inhibited the binding of ACE2 to the spike and all reported variants across all three timepoints (**Figure 3**).

### Association between breast epithelium permeability and anti-RBD antibodies in milk

We assessed Na:K ratios in milk as a measure of breast-epithelium permeability. First, to assess the precision of our assay, we randomly selected archived milk from five participants for whom we had extra aliquots. Triplicate measures of Na:K had coefficients of variation below 5% for milk from all five participants (**Figure S3**).

**Figure 4:**
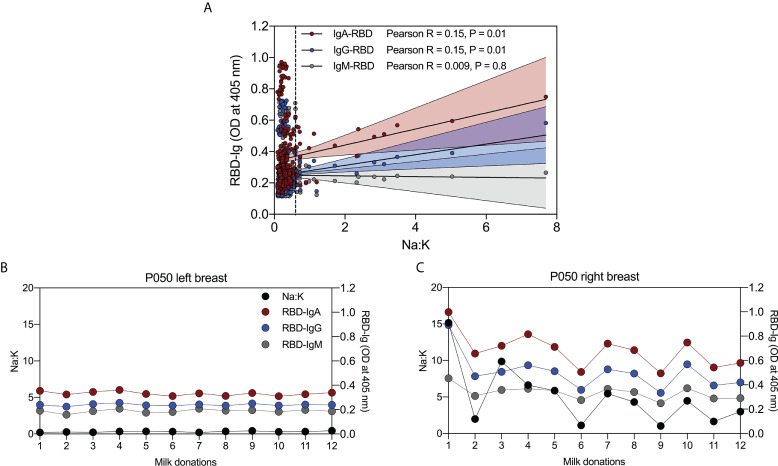
Abnormally high permeability of breast epithelium is associated with levels of RBD-reactive antibodies in milk. **(A)** Weak positive correlation between Na:K and RBD-IgA, and -IgG, and no correlation between Na:K and RBD-IgM in serial milk from 30 women with COVID-19. **(B)** Relationship between Na:K and RBD-IgA, -IgG, and -IgM in milk from the left breast and **(C)** right breast from a single woman with COVID-19. Vertical dashed line in **(A)** indicates an Na:K = 0.6.

Twenty-seven percent of women had Na:K ratios > 0.6, a cut-off frequently used to define above-normal permeability (19). As shown in **Figure 4A** there was a weak but statistically significant association between Na:K (mean ratio of left and right breasts) and levels of anti-RBD-IgA (Pearson R = 0.15, P = 0.01) and RBD-IgG (Pearson R = 0.15, P = 0.01). However, these associations were reliant on the inclusion of samples from a single participant, P050. Removal of data from P050 eliminated the significant associations between Na:K and anti-RBD-IgA (Pearson R = 0.02, P = 0.6) and IgG (Pearson R = 0.04, P = 0.5).

Closer examination of the data from P050 revealed significant but weak associations between Na:K (mean ratio of left and right breasts) and anti-RBD-IgA and IgG (data not shown). To further investigate this relationship, we measured anti-RBD IgA, IgG and IgM in serial milk samples from the left and right breasts, separately. Na:K ratios in milk from the left breast (black circles) were similar at all 12 timepoints (range = 0.2 – 0.44); likewise, the three anti-RBD antibodies were steady across the timepoints (**Figure 4B**). In contrast, results from the right breast show fluctuating Na:K ratios (range = 1.03 – 15.17) and that antibody levels track closely with Na:K ratios across all time points (anti-RBD IgA; Pearson R = 0.89, *P*< 0.0001, anti-RBD IgG; Pearson R = 0.91, *P*< 0.0001, and anti-RBD IgM; Pearson R = 0.90, *P*< 0.0001) (**Figure 4C**).

### SARS-CoV-2-reactive IgG and IgA were detected in stool from only one of the infants

We next assessed anti-RBD IgA, IgG and IgM levels in ethanol-preserved stool samples from 26 infants of mothers with COVID-19. Stool samples from the second collection were provided for 11 of the infants for a total of 37 stool samples. We detected total IgA and IgG in all ethanol-preserved infant stool samples, and total IgM in most samples (**Figure S4A**). In contrast, stool from only one infant had RBD-reactive IgG and IgA, and this reactivity was apparent only at day 35 (**Figure S4B**).

### Breastmilk from women with COVID-19 contain T cells expressing mucosal-homing markers

**Figure 5:**
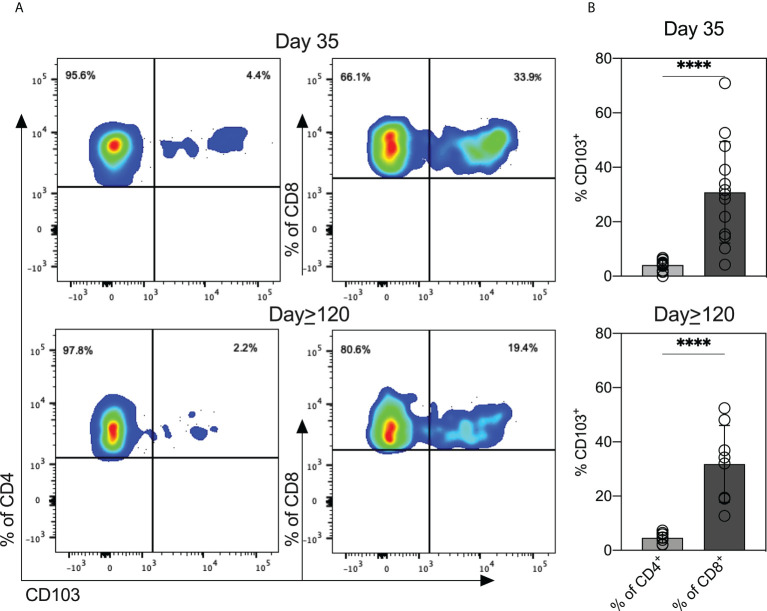
T cells in breastmilk from SARS-CoV-2-infected women display high levels of mucosal-homing marker. **(A)** Scatter plots from one representative participant showing CD103 expression on CD4^+^ and CD8^+^ T cell populations in milk obtained at the two indicated timepoints. **(B)** Expression of CD103 on CD4^+^ and CD8^+^ in breastmilk cells (*n* = 13 Day 35; *n* = 8 Day≥120). Independent *t*-tests were performed to analyze differences between CD103 expression within CD4^+^ and CD8^+^ T cells. ****P<0.0001.

We measured levels of ten cytokines in milk expressed at three timepoints (*outlined in the Methods*). Thirty milk samples were analyzed at Day 1 and Day 34, and 16 samples were analyzed at Day≥120. As a control we included the cytokine levels in milk provided by women without COVID-19 from an earlier study (n = 26) (11). Except for IL-2, median levels of cytokines were similar in milk expressed across the three timepoints (**Figure S5**). To expand our understanding of the T cell responses to SARS-CoV-2 present in milk, we examined cryopreserved cells by flow cytometry. The distribution of CD4^+^ and CD8^+^ T cells was similar in the D35 and D≥120 samples (**Figure S6A**). We investigated the expression of CD103^+^ mucosal-homing marker on CD4^+^ and CD8^+^ T cells. The CD8^+^ population expressed higher levels of CD103 than did the CD4^+^ population, in both the D35 milk (34% CD8^+^/CD103^+^ vs 4.4% CD4^+^/CD103^+^, *P*< 0.0001) and D≥120 milk (19.4% CD8^+^/CD103^+^ vs 2.2% CD4^+^/CD103^+^, *P*< 0.0001) (**Figure 5**). However, the percentages of CD4^+^/CD103^+^ and CD8^+^/CD103^+^ were similar between the D35 and D≥120 samples (**Figure S6B**).

### Breastmilk from COVID-19-recovered women is enriched for antigen-experienced T cells

**Figure 6:**
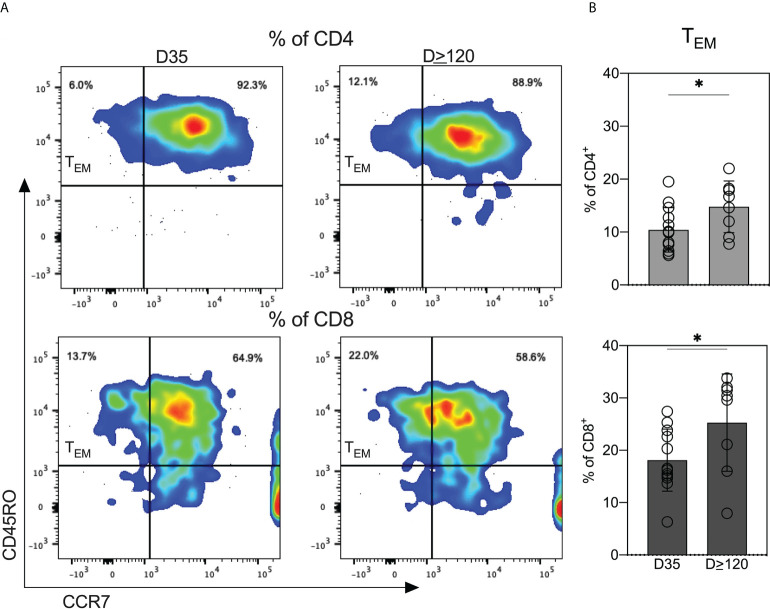
Breastmilk obtained from COVID-19-recovered women is enriched for antigen-experienced T cells. **(A)** Scatter plots from one representative participant showing the distribution of effector memory (T_EM_; CD45RO^+^/CCR7^-^) and central memory (T_CM_; CD45RO^+^/CCR7^-^) T cells in milk provided at the two indicated timepoints. **(B)** Comparison of T_EM_ within CD4^+^ and CD8^+^ T cells between milk obtained at the two indicated timepoints. Independent *t* tests were performed to analyze differences in T_EM_ expression within CD4^+^ and CD8^+^ T cells between the day 35 and day ≥120 samples. *P<0.05.

Next, we investigated the expression of memory T cell subsets in cryopreserved cells from D35 and D≥120 breastmilk. At both timepoints, nearly all CD4^+^ and CD8^+^ T cells were CD45RO^+^ antigen experienced (**Figure 6A**). We determined the percentages of CD45RO^+^/CCR7^+^ (T_CM_) and CD45RO^+^/CCR7^-^ (T_EM_). The majority of both CD4^+^ and CD8^+^ populations were T_CMs_, independent of time of milk expression (**Figure 6A**). Interestingly, the percentages of T_EM_ (CD45RO^+^/CCR7^-^) on both CD4^+^ and CD8^+^ increased significantly from D35 to D≥120 (**Figure 6B**).

## Discussion

Our comprehensive study provides the kinetics of anti-RBD antibodies in milk from women with COVID-19. In the setting of SARS-CoV-2 infection, we are the first to examine viral-elicited T cells in these milk samples. This is an important and unexplored area of COVID-19 immunity. Studies have shown that gut-homing lymphocytes transferred to the infant *via* breastmilk can survive in the infant digestive tract and seed gut tissues (12, 21–23). To expand our understanding of the cellular responses in the milk of women with a prior SARS-CoV-2 infection, we assessed the levels of T cell subsets in milk expressed around 35 and ≥120 days after a positive COVID-19 test. We specifically assessed mucosal-homing (CD103^+^) and memory T cell subsets (CD45RO^+^CCR7^+/-^) in the milk samples. We find that breastmilk T cells have high expression of CD103 and are nearly uniformly CD45RO^+^. CD103 is an α_E_β_7_ integrin expressed on intra-epithelial lymphocytes in mucosal sites (24). The high expression of mucosal-homing markers in breastmilk T cells suggests that these cells likely arise from a tissue-resident population. We detected higher expression of CD103 on CD8^+^ T cells compared to CD4^+^ T cells, consistent with published studies not in the setting of COVID-19 (14, 15, 25). This is expected as the intra-epithelial lymphocytes in the small intestine are almost exclusively CD8^+^ (26), leading to more CD8^+^/CD103^+^ T cells in milk compared to CD4^+^/CD103^+^ T cells. We also detected increased T_EM_ on both CD4 and CD8 populations in cells from milk expressed at the four-month follow-up. T_EMs_ are a subset of memory cells that can perform both effector and memory functions (27). That more CD8^+^ T cells were also CD103^+^ suggests that milk from COVID-19-recovered women contains increased T_EM_ populations having both cytotoxic and memory phenotypes. Our results indicate that the mucosal-homing, effector-memory T cells are transferred from milk to the breastfed infant. This may have significance as some previously published studies suggest that the cells can remain viable and seed the infant gastrointestinal tract (12, 21–23), thereby providing passive protection.

Findings from our longitudinal study are consistent with published data on the humoral immune response in the milk of women with COVID-19 (6–8) and show: (1) that the milk of most women with COVID-19 have anti-RBD IgA and IgG; (2) that RBD-specific antibodies are present in the milk of most women for at least four-months after a COVID-19 positive test; and (3) the presence of a robust RBD-specific IgA response in milk but a lack thereof in circulation. We detected RBD-specific IgA, IgG, and IgM in milk obtained between three and 33 days after a positive COVID-19 test in 73%, 90% and 50% of the women, respectively, reinforcing the finding that SARS-CoV-2 infection elicits a robust humoral immune response in the breastmilk of lactating women (4, 5, 7–10, 28). Our study also shows that the median levels of RBD-specific IgA and IgG in milk expressed at the four-month follow-up were similar to levels detected weeks after infection. Additionally, COVID-19 was associated with a rapid, and long-lasting IgA response in the milk from most women. Our longitudinal analysis showed that RBD-specific IgM levels decreased in milk expressed at the four-month follow-up. Indeed, prior studies have observed that anti-SARS-CoV-2 IgM antibodies decayed while IgA and IgG remained stable in serum and saliva (29, 30). We observed that the immune response in milk is IgA and IgG (4, 7–9), in contrast to the predominantly IgG-driven humoral response in the milk from women receiving an mRNA-based COVID-19 vaccine (6, 11, 31, 32). This is likely because SARS-CoV-2 is acquired mucosally whereas mRNA-based vaccines are administered intramuscularly. Moreover, the detection of a robust IgA response in the milk of infected women and a lack thereof in their circulation is expected given the entero-mammary link. Milk IgA is produced by mammary gland B cells that have migrated from the intestine through the entero-mammary pathway (13) therefore nearly all the milk IgA is in the secretory form. The secretory component of IgA ensures its effective transfer to the infant and its survival in the gastric environment (8, 33).

RBD-antibodies in milk neutralized the SARS-CoV-2 spike protein and four of its variants. Compared to the control milk samples (pre-COVID-19 and pre-vaccine), milk provided immediately after a positive test had significantly increased (*P*< 0.05) neutralizing ability against the spike and its variants (**Figure 3**). Importantly, whether an IgA or an IgG response is dominant, milk from both infected women and vaccinated mothers in our prior study (11) neutralized the spike and its variants.

Infants born to mothers neither vaccinated against COVID-19 nor exposed to the virus lack an immune response to SARS-CoV-2. For these infants, vertical transmission of anti-SARS-CoV-2 antibodies *via* breastmilk provides the only protection. We previously reported that stool samples from 30% and 33% of breastfed infants of mothers vaccinated while lactating are positive for anti-RBD IgA and IgG, respectively (11). In the present study, we measured the levels of anti-RBD antibodies in stool from infants of mothers diagnosed with COVID-19 while lactating. Anti-RBD IgA and IgG were detected in stool from only one infant. Detection of anti-SARS-CoV-2 antibodies in infant stool indicates protection at mucosal sites, and therefore the variability observed among infants warrants further study. Detection of antibodies in infant sera of mothers vaccinated or exposed to the virus while lactating would suggest systemic protection against SARS-CoV-2 and only a handful of studies have addressed this aspect of vertical transmission. Among the limited literature, three studies reported that anti-SARS-CoV-2 antibodies were not detected in infant sera of mothers vaccinated while lactating (sample sizes = 8, 5, and 21) (31, 34, 35), and one study reported that anti-SARS-CoV-2 IgG was detected in the sera of 23% (3 of 13) of breastfed infants of mothers vaccinated after delivery (36). In contrast, it is clear that transplacental transmission after maternal vaccination or infection results in detectable levels of anti-SARS-CoV-2 antibodies in infant sera (34, 37–40). It is important to keep in mind that COVID-19 could manifest as an asymptomatic infection, both among infants and among lactating women. Therefore, it is possible that infants of mothers vaccinated after delivery could have acquired anti-SARS-CoV-2 antibodies transplacentally following a maternal asymptomatic infection during pregnancy. It also is important to recognize the small samples sizes of the studies conducted to date. Taken together, it seems that further studies are necessary to better understand vertical transmission of anti-SARS-CoV-2 antibodies, specifically IgG in infant sera, and future studies should include a larger population with detailed information on exclusivity of breastfeeding.

During lactation, tight junctions in the breast epithelium close the paracellular pathway limiting communication between the maternal bloodstream and the mammary gland (41, 42). Development and maintenance of these tight junctions and the consequent limited permeability are important, for milk synthesis, composition, and secretion (41, 43). Breakdown of tight junctions and elevated breast epithelium permeability occurs during milk accumulation and with inflammatory and immune responses and is associated with an influx of antibodies to breastmilk (41). We asked whether the permeability of the breast epithelium was associated with anti-RBD antibodies in the milk of women with COVID-19. Although 27% of women had at least one milk sample with elevated breast epithelium permeability, as defined by a Na:K ratio of ≥ 0.6, there was no overall significant relationship between permeability and anti-RBD antibody levels. A significant relationship was shown for one participant who had elevated permeability in only one breast; indeed, her antibody levels tracked closely with Na:K.

Published data indicate that elevated breast epithelium permeability is associated with increased interleukin (IL)-8, IL-1β, and TNFα (42–45). In the present study, we also observed significantly increased levels of cytokines in the milk of participant P050 when Na:K ratios were high (**Figure S7**). The cytokines IL-8, IL-1β, and TNFα increase expression of polymeric immunoglobulin receptor (pIgR) on epithelial cells, which is responsible for the transcytosis of locally synthesized IgA across epithelial cells (44, 45). However, IgG and IgM are predominantly transferred to milk from the circulation (41). Given the importance of milk-derived antibodies and cytokines in the development of the infant gut and immune system (12, 13), the extent to which increased breast permeability influences antibody and cytokine levels in milk from women with a prior SARS-CoV-2 infection warrants further study.

Collectively, we have shown that antibodies in milk from women with a prior SARS-CoV-2 infection can neutralize the spike complex. Importantly, we show that breastmilk T cells are enriched for mucosal memory T cells, further emphasizing the passive protection to infants against SARS-CoV-2 conferred by nursing infected mothers.

## Data availability statement

The original contributions presented in the study are included in the article/**Supplementary Material**. Further inquiries can be directed to the corresponding author.

## Ethics statement

The studies involving human participants were reviewed and approved by University of Massachusetts IRB protocol 2075. The patients/participants provided their written informed consent to participate in this study.

## Author contributions

KA, BP, and VN conceptualized and designed the study. KA, SN, and AS developed the REDCap database and recruited participants. VN, KA, BP, JT, AB, and SS contributed to the design of experiments. DA developed the anti-RBD ELISAs and provided RBD. VN, AS, and RB conducted ELISAs and MSD assays. VN conducted the flow cytometry, analyzed data, and wrote the first version of the manuscript. All authors read and approved the final version of the manuscript.

## Funding

This research was supported by UMass-Amherst Seed Funding and NIH grant 5R01CA230478-02 to KA, NIH grant R24OD021485 to DA, and UMass-Amherst Graduate Dissertation Research Award to VN.

## Conflict of interest

The authors declare that the research was conducted in the absence of any commercial or financial relationships that could be construed as a potential conflict of interest.

## Publisher’s note

All claims expressed in this article are solely those of the authors and do not necessarily represent those of their affiliated organizations, or those of the publisher, the editors and the reviewers. Any product that may be evaluated in this article, or claim that may be made by its manufacturer, is not guaranteed or endorsed by the publisher.
