# Supplementary material for: Durable antibody and effector memory T cell responses in breastmilk from women with SARS-CoV-2
Source: Front Immunol. 2022 Sep 12;13:985226. doi: 10.3389/fimmu.2022.985226 (PMC9512087; doi:10.3389/fimmu.2022.985226)
Supplement: Supplementary file 1 [file DataSheet_1.docx]

**Durable antibody and effector memory T cell responses in breastmilk from women with SARS-CoV-2.**

Vignesh Narayanaswamy, *et al*

**Supplementary Figures**

**Figure S1**

**
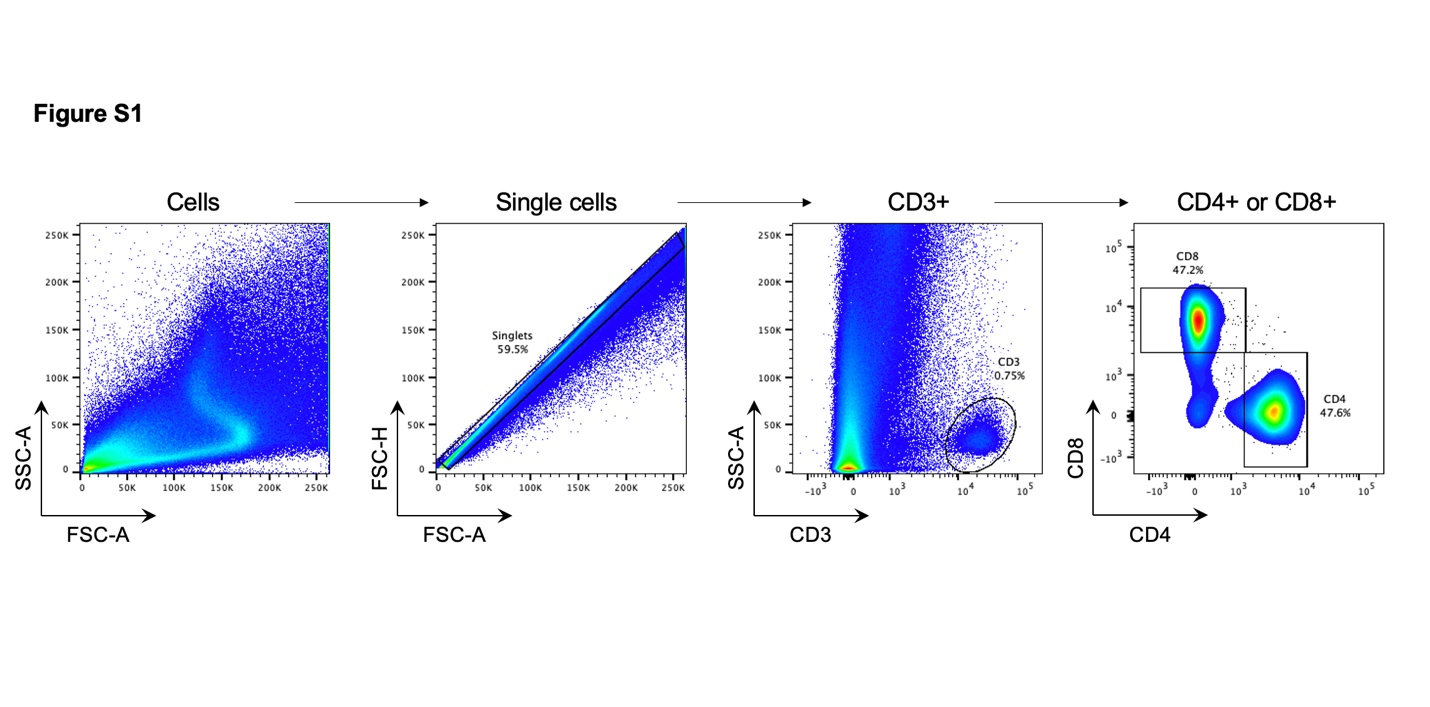
**

**Figure S1. Gating strategy for phenotyping cells in milk from who women who previously tested positive for SARS-CoV-2**. The cells were stained with the panel shown in **Table S1**.

**Figure S2**

**Figure S2. Anti-receptor binding domain (RBD)-specific immunoglobulin (Ig)G detected in dried blood spots (DBS) from women who previously tested positive for SARS-CoV-2**. Comparison of anti-RBD-IgA, IgG, and IgM in DBS samples between the indicated timepoints. Differences were analyzed using independent *t* tests. **P* < 0.05

**Figure S3**

**Figure S3. Performance evaluation of the Medica EasyLyte Na:K Analyzer.** Precision of triplicate measures in milk samples from five participants determined with ion-selective electrodes.

**Figure S4**

**
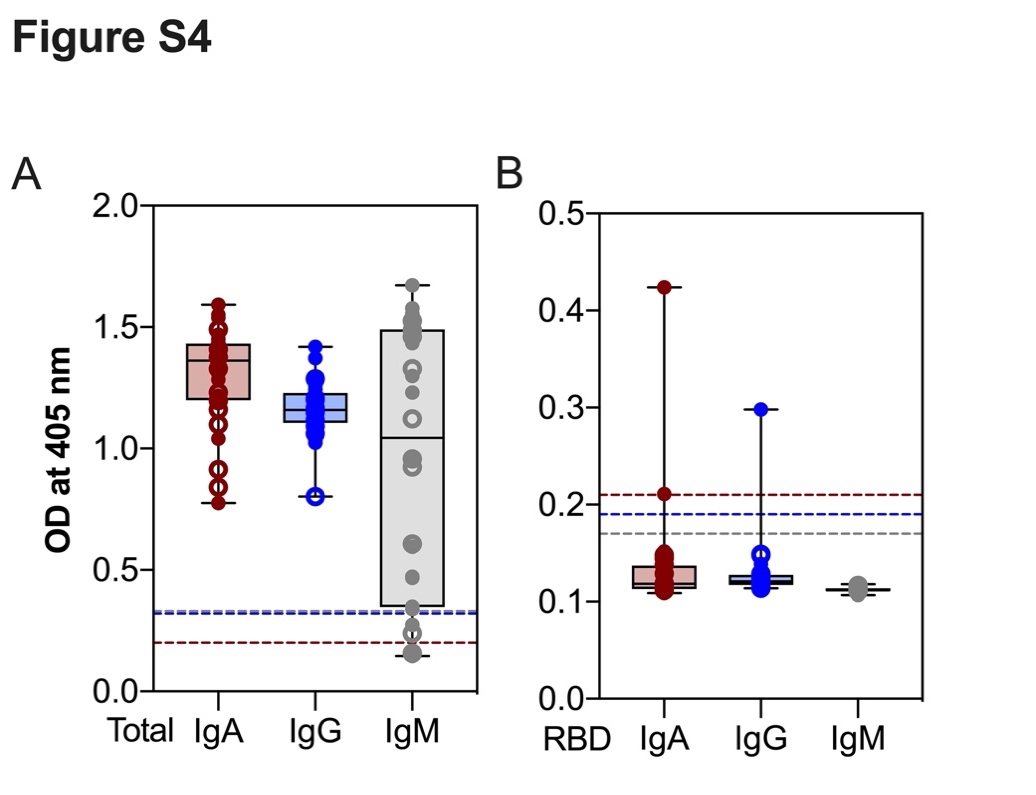
**

**
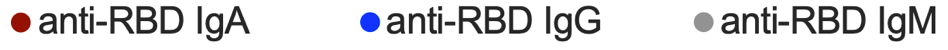
**

**Figure S4. SARS-CoV-2 RBD-specific IgA and IgG detected in stool sample from only one infant of a mother with COVID-19.** Levels of total immunoglobulins **(A)** and RBD-specific IgA, IgG, and IgM (**B)** in stool from infants of mothers with COVID-19. Filled circles indicate Day 34 samples (n = 26) and open circles indicate Day >120 samples (n = 11). Horizontal dashed lines indicate positive cut-off values.

**Figure S5**

**Figure S5. Only IL-2 showed a significant increase over time in milk obtained from women who previously tested positive for SARS-CoV-**2. Milk was assayed at the three indicated timepoints. Day 1: the first milk samples expressed after enrollment (*n* = 30), Day 34: the last milk samples (of the first mailed set) expressed after enrollment (*n* = 30), Day>120: samples collected after at least four months since the positive COVID-19 PCR test (*n* = 15). The control data are from our previous publication (10) in which women who had not had COVID-19 provided milk prior to vaccination. Horizontal lines in each bar indicate median concentration (pg/mL).

**Figure S6**

**Figure S6. Percentages of CD4, CD8, and mucosal-homing cells within CD4 and CD8 populations did not change as a function of time**. (**A**) Distribution of CD4+ (*left*) and CD8+ (*right*) in cells within milk expressed at the two timepoints. (**B**) Expression of CD103 on CD4+ (l*eft*) and CD8+ (*right*) in cells within milk expressed at the two timepoints (*n* = 13 Day 35; *n* = 8 Day>120).

**Figure S7**

**Figure S7. Significantly elevated levels in seven of eight cytokines in the milk from the right breast compared to milk from the left breast from participant P050.** Each filled circle indicates milk provided at a single timepoint (12 in total). Horizontal lines in each box indicate median concentration (pg/mL), ****P* < 0.001, *****P* < 0.0001.
