# Supplementary material for: Durable antibody and effector memory T cell responses in breastmilk from women with SARS-CoV-2
Source: Front Immunol. 2022 Sep 12;13:985226. doi: 10.3389/fimmu.2022.985226 (PMC9512087; doi:10.3389/fimmu.2022.985226)
Supplement: Supplementary file 3 [file Table_2.docx]

| **Table S2**. Flow cytometry panel. | | | | |
| --- | --- | --- | --- | --- |
| **Marker** | **Clone** | **Fluorochrome** | **Catalog #** | **Dilution** |
| CD3 | OKT3 | PE/Cy7 | 317333 | 1:200 |
| CD4 | RPA-T4 | FITC | 300505 | 1:50 |
| CD8 | SK1 | PerCP/Cy5.5 | 344709 | 1:50 |
| CCR7 | G043H7 | APC | 353213 | 1:50 |
| CD45RO | UCHL1 | PE | 304205 | 1:50 |
| CD103 | Ber-ACT8 | BV421 | 350213 | 1:50 |

*All antibodies were purchased from BioLegend*.
